# Non-response to ACE items is associated with demographic variables and health indicators in the 2021 Behavioral Risk Factor Surveillance System

**DOI:** 10.1016/j.pmedr.2024.102749

**Published:** 2024-05-03

**Authors:** Timothy J. Grigsby, Madalyn Larson, Andrea Lopez, Sarah Sharmin, Ying Guo, Myriam Forster, Sheniz Moonie

**Affiliations:** a Department of Social and Behavioral Health, University of Nevada, Las Vegas, United States; b Department of Social and Behavioral Health, University of Nevada, Reno, United States; c Department of Epidemiology and Biostatistics, University of Nevada, Las Vegas, United States; d Department of Demography, University of Texas at San Antonio, United States; e Department of Environmental and Occupational Health, University of Nevada, Las Vegas, United States; f Department of Health Sciences, California State University, Northridge, United States

**Keywords:** ACE, BRFSS, Non-response, Smoking, Obesity, Health

## Abstract

**Background:**

Adverse childhood experiences (ACE) encompass traumatic events occurring before age 18, with lasting impacts on health. While ACE disclosure is important for understanding these effects, some individuals decline to respond to ACE-related survey items due to sensitivity, privacy concerns, or psychological distress. This study explores the relationship between non-response to ACE items and health outcomes, shedding light on the implications for those who choose not to disclose.

**Methods:**

We performed a secondary analysis of the 2021 Behavioral Risk Factor Surveillance System (BRFSS)—a national telephone survey querying health behaviors and conditions. Sociodemographic factors, ACE exposure, and non-response to ACE items were analyzed.

**Results:**

Individuals who decline to respond to ACE items exhibit similar patterns of health behaviors and conditions as those reporting ACE exposure. Non-response is linked to both healthier behaviors (lifetime HIV testing) and riskier behaviors (higher odds of smoking and e-cigarette use). Moreover, non-responders have higher odds of being underweight or obese, experiencing concentration difficulties, reporting poor self-rated health, and reporting multiple health diagnoses including depression, diabetes, high blood pressure, heart attack, and stroke.

**Conclusions:**

The study underscores the need to address health disparities associated with ACE, regardless of disclosure status. Healthcare interventions should target respondents and non-respondents of ACE screeners, tailoring strategies to promote healthier coping mechanisms and mitigate maladaptive behaviors. These results emphasize the importance of trauma-informed care, early intervention, and targeted public health initiatives for individuals affected by ACE, irrespective of their disclosure choices.

## 1 Introduction

Adverse childhood experiences (ACE) are commonly described as a set of highly correlated and cumulative family-based traumatic experiences that occur before age 18 and include abuse (psychological/verbal, physical, and sexual), neglect, and household dysfunction (e.g., parental intimate partner violence, household member substance misuse and mental illness, and household member incarceration) (Felitti et al., 1998). ACE impact health through complex mechanisms that can disrupt normal neurodevelopmental processes leading to alterations in stress response systems, cognitive function, and emotional regulation (Danese and McEwen, 2012, Rudenstine et al., 2019). The long-term consequences of these perturbations on biological systems—including the immune, endocrine, and nervous systems—contribute to the emergence of a myriad of health conditions (Danese & Lewis, 2017).

The social and environmental contexts in which ACE occur are crucial in understanding the relationship between adversity and health. Socioeconomic disadvantage, community violence, inadequate access to healthcare, and limited social support interact with ACE and shape the long-term health consequences experienced by individuals (Finkelhor et al., 2013). Documenting the relationship between ACE and health outcomes at the population level is critical. First, ACE are prevalent, with estimates suggesting 64 % of the U.S. adult population report at least one ACE and 17.5 % report four or more ACE (CDC, 2023). Second, epidemiological studies have consistently shown that ACE are not isolated incidents, but rather co-occur and have cumulative effects (Felitti et al., 1998). Finally, the influence of ACE extends beyond childhood, and acknowledging the significance of protective factors that bolster resilience is crucial in understanding the sequelae of ACE on individuals' well-being throughout the lifespan (Anda et al., 2006, Hajat et al., 2020).

Among studies examining physical health outcomes using the Behavioral Risk Factor Surveillance System (BRFSS), evidence strongly suggests that compared to individuals who do not have a history of ACE, persons who experience these adversities have disproportionately high physical and mental health problems in adulthood (Hall et al., 2020, Merrick et al., 2019, Mwachofi et al., 2020, Sonu et al., 2019) and are more likely to self-report poor overall health and reduced health-related quality of life (Crouch et al., 2017, Downing et al., 2021). Research with the BRFSS data has also demonstrated the impact of ACE on behavioral health. For instance, ACE exposure is associated with substance use (Alcalá et al., 2016, Lee and Chen, 2017, Loudermilk et al., 2018), Human immunodeficiency virus (HIV) risk behaviors (Fang et al., 2016), and diet and exercise practices (Mendoza et al., 2023). Cumulatively, ACE have been shown to have long-lasting effects on multiple health indicators, highlighting the need for early intervention and prevention strategies to address the negative health impact of ACE throughout the lifespan.

As a result of epidemiological research on ACE and health, calls for universal screening of ACE to identify individuals who may be at a heightened risk for negative health outcomes has gained attention across sectors. Incorporating ACE screening practices in diverse settings, such as schools and medical offices, offers several benefits. Early detection and intervention can ensure timely support for individuals affected by ACE through the implementation and referral to trauma-informed programming, fostering enhanced collaboration and coordination across providers, and promoting comprehensive care that addresses the interconnected physical, emotional, and social aspects of health. Additionally, gathering ACE data in these settings allows for targeted interventions, policy development, and resource allocation that can better serve individuals and communities impacted by childhood adversity. One example of such efforts is the “ACE Aware” initiative (ACEs Aware. (n.d.), California Department of Health Care Services, n.d.), a comprehensive program implemented in medical settings to systematically screen patients for ACE exposure. This program aims to increase awareness, improve identification, and enhance appropriate responses to ACE. Through early detection, healthcare professionals can provide timely interventions, support, and referrals to address the underlying trauma in an effort to mitigate related health outcomes. In educational settings, trauma-informed schools (Wiest-Stevenson & Lee, 2016) recognize the prevalence of ACE among students and aim to create a supportive and responsive educational environment. These schools incorporate trauma-informed practices into various aspects of school life—including classroom management, disciplinary approaches, social-emotional learning, and support services.

Surveys and screening tools have become increasingly valuable for developing and implementing targeted ACE-based interventions and incorporating trauma-informed approaches into existing programming, although it is important to acknowledge that some individuals may choose to decline or omit responses to ACE questions. Declining to respond is especially important considering surveys of adults rely on retrospective reports which have been challenged with respect to their validity and accuracy of recalling traumatic childhood events (Hardt & Rutter, 2004). There are several reasons why non-response may occur, and understanding these factors is critical for interpreting and addressing potential limitations of ACE survey research and screening efforts. First, individuals may decline to respond to ACE items due to the sensitive and personal nature of the experiences that can elicit feelings of shame, fear, or discomfort when recalling or disclosing such events. Consequently, individuals may opt to withhold their responses to protect their privacy, maintain emotional boundaries, or avoid revisiting painful memories. Second, cultural or societal factors can amplify any inclination to withhold information, driven by stigma associated with discussing personal hardships or societal norms that discourage seeking help. Moreover, individuals who have experienced ACE may struggle with trust and may perceive surveys or screening measures as intrusive or judgmental. Relatedly, the fear of potential repercussions of disclosure, such as legal consequences or child protective services involvement, could dissuade individuals from providing responses. Concerns about confidentiality, particularly if they have not been clearly communicated or confirmed, can also contribute to hesitancy in revealing sensitive information.

The current study aimed to explore patterns in the proportion of U.S. adults who declined to respond to ACE items on the 2021 BRFSS survey by (a) examining sociodemographic characteristics associated with non-response, and (b) the relationship between the amount of declined ACE items and health indicators. We present results for cumulative ACE exposure for comparison. We hypothesized that declining to answer more ACE items would be associated with a higher odds of reporting negative health outcomes akin to previously observed associations with cumulative ACE exposure.

## 2 Methods

### 2.1 Data source

This study analyzed de-identified data collected as part of the 2021 Behavioral Risk Factor Surveillance System (BRFSS), a publicly available U.S. government data set. The BRFSS survey includes questions about health risk behaviors, chronic diseases, and healthcare access (CDC - BRFSS, 2021). Eligible participants were required to be aged 18 years of age or older, residents of either the US or its territories, not being incarcerated or in assisted living, and equipped with either a cellular device or a landline. In 2021, the survey was administered across all fifty states, the District of Columbia, Guam, Puerto Rico, and the US Virgin Islands. Interviews were conducted throughout every month, ensuring coverage seven days a week during the daytime hours. Of 438,693 BRFSS respondents, a subset of 358,899 respondents completed the full interview, and 58,278 respondents participated in the optional ACE module offered in eleven states (Alabama, Arkansas, Iowa, Mississippi, Nevada, New Hampshire, North Dakota, Oregon, South Carolina, Virginia, and Wisconsin). In a supplemental analysis, we replicated analyses with 128,496 respondents who completed the ACE module in the 2020 BRFSS. This secondary analysis was deemed exempt by the University of Nevada, Las Vegas Institutional Review Board.

## 3 Measures

*Adverse Childhood Experiences (ACE)*. Participants self-reported whether they had experienced any of 11 ACE items before the age of 18. The adversities covered two general categories: abuse (physical and sexual) and household dysfunction (e.g., exposure to drugs and alcohol in the home, household member incarceration). Two variables were calculated to estimate exposure and non-response (i.e., declined to respond). The number of “yes” responses was summed to calculate the total ACEs score, ranging from 0 to 11. Similarly, the number of “don’t know” and “prefer not to answer” were coded 1 and summed (range: 0–11). To facilitate analyses, the continuous ACE score was categorized into three groups based on conventions in the literature: individuals with 4 or more ACEs, those with 1–3 ACEs, and those with no ACEs (reference group). Likewise, we coded declined to respond as 0 missing, 1–3 missing, and 4 or more missing.

*Sociodemographic Variables*. We examined ACE non-response over three demographic characteristics included in the BRFSS survey including age (18–24, 25–34, 35–44, 45–54, 55–64, 65 and older), sex assigned at birth (female, male), and race/ethnicity (non-Hispanic Asian, non-Hispanic Black, non-Hispanic White, Hispanic (any), Other/Mixed, Unknown).

*Health Indicators*. Three categories of health indicators were examined in relation to ACE non-response and ACE exposure. All variables were coded as no (0; reference) and yes (1). Health behaviors included past month smoking, e-cigarette use, smokeless tobacco use, binge drinking, heavy drinking, exercise, and ever being tested for human immunodeficiency virus (HIV). Health conditions included underweight status (BMI < 18), obese status (BMI > 30), difficulty concentrating or remembering, and self-rated health of “fair” or “poor” (compared to “good” or “very good” coded 0). Health diagnoses included having ever received a diagnosis of depression, diabetes, heart attack, high blood pressure, high cholesterol, or stroke.

## 4 Analytic plan

First, the prevalence of individual ACE exposure and non-response was estimated. Second, tetrachoric correlations of individual ACE by exposure and non-response were calculated with Bonferroni correction for multiple comparisons. Third, we assessed patterns of exposure and non-response by demographic characteristics and present weighted estimates. Fourth, we examined the association between demographic characteristics and rate of ACE exposure and non-response using separate negative binomial regression models with results presented as Incident Rate Ratios (IRR) with 95 % confidence intervals (95 % CI). Fifth, we used logistic regression models to estimate relationships between cumulative missing ACE items (i.e., declined to respond) with various health indicators included in the BRFSS adjusting for age, sex, and race/ethnicity. Associations with cumulative ACE exposure are presented for comparison. Results are reported as odds ratios (OR) with 95 % confidence intervals. In all analyses, we employed survey weights and design-based variance estimators to account for the complex survey design of the BRFSS using the “svy:” command in Stata version 18 (Stata Corp., 2023).

## 5 Results

The unweighted analytic sample of 58,278 respondents from 11 U.S. States (Alabama, Arkansas, Iowa, Mississippi, Nevada, New Hampshire, North Dakota, Oregon, South Carolina, Virginia, and Wisconsin). The weighted sample was 51.8 % female and had a median age of 45–54 years old. As shown in Table 1, the weighted prevalence of specific ACE exposure in this sample varied between 5.48 % (forced sex) and 36.11 % (verbal abuse). Rates of declined to answer specific ACE items varied between 1.95 % (household incarceration) and 4.61 % (parental divorce). Tetrachoric correlations with Bonferroni correction (Table 2) showed statistically significant moderate to large correlations between specific ACE exposures (r = 0.3145 to 0.9312, p’s < 0.001) and declined to answer ACE items (r = 0.7714 to 0.9856, p’s < 0.001).

**Table 1:** Weighted prevalence of ACE Exposure and Declined to Respond by item in the 2021 Behavioral Risk Factor Surveillance Survey (unweighted n = 58,278).

Item	% Exposed	% Declined
Did you live with anyone who was depressed, mentally ill, or suicidal?	20.93	2.82
Did you live with anyone who was a problem drinker or alcoholic?	24.99	2.24
Did you live with anyone who used illegal street drugs or who abused prescription medications?	12.49	2.43
Did you live with anyone who served time or was sentenced to serve time in a prison, jail, or other correctional facility?	9.59	1.95
Were your parents separated or divorced?	29.70	4.61
How often did your parents or adults in your home ever slap, hit, kick, punch or beat each other up?	16.94	3.96
Not including spanking, (before age 18), how often did a parent or adult in your home ever hit, beat, kick, or physically hurt you in any way?	23.65	3.32
How often did a parent or adult in your home ever swear at you, insult you, or put you down?	36.11	3.47
How often did anyone at least 5 years older than you or an adult, ever touch you sexually?	11.90	3.43
How often did anyone at least 5 years older than you or an adult, try to make you touch them sexually?	9.11	3.38
How often did anyone at least 5 years older than you or an adult, force you to have sex?	5.48	3.30

Note: Prevalence estimates were calculated using the “svy: tab” command in Stata version 16.

**Table 2:** Tetrachoric associations between exposure to individual ACE (above dashed line) and declined to answer individual ACE (below dashed line).

	1	2	3	4	5	6	7	8	9	10	11
1. Did you live with anyone who was depressed, mentally ill, or suicidal?	−-	0.4953	0.6127	0.4833	0.3763	0.4798	0.4487	0.5537	0.4430	0.4383	0.4344
2. Did you live with anyone who was a problem drinker or alcoholic?	0.9468	−-	0.6152	0.5594	0.4152	0.5918	0.4228	0.4959	0.3841	0.3805	0.3876
3. Did you live with anyone who used illegal street drugs or who abused prescription medications?	0.9421	0.9688	−-	0.7415	0.4668	0.5022	0.4250	0.4978	0.4218	0.4395	0.4367
4. Did you live with anyone who served time or was sentenced to serve time in a prison, jail, or other correctional facility?	0.9511	0.9767	0.9768	−-	0.4922	0.5095	0.3925	0.4327	0.3784	0.3958	0.4039
5. Were your parents separated or divorced?	0.8176	0.8772	0.8728	0.9067	−-	0.4791	0.3145	0.3648	0.3193	0.3211	0.3256
6. How often did your parents or adults in your home ever slap, hit, kick, punch or beat each other up?	0.8531	0.9033	0.8964	0.9285	0.7736	−-	0.6329	0.6116	0.4482	0.4450	0.4622
7. Not including spanking, (before age 18), how often did a parent or adult in your home ever hit, beat, kick, or physically hurt you in any way?	0.8758	0.9179	0.9112	0.9372	0.8003	0.9080	−-	0.6452	0.4500	0.4510	0.4724
8. How often did a parent or adult in your home ever swear at you, insult you, or put you down?	0.8579	0.9072	0.9000	0.9250	0.7727	0.8670	0.9199	−-	0.4606	0.4605	0.4794
9. How often did anyone at least 5 years older than you or an adult, ever touch you sexually?	0.8585	0.9049	0.8965	0.9274	0.7714	0.8489	0.8835	0.8644	−-	0.9312	0.8792
10. How often did anyone at least 5 years older than you or an adult, try to make you touch them sexually?	0.8634	0.9082	0.8999	0.9294	0.7763	0.8473	0.8803	0.8643	0.9882	−-	0.8739
11. How often did anyone at least 5 years older than you or an adult, force you to have sex?	0.8673	0.9096	0.9046	0.9326	0.7836	0.8527	0.8875	0.8685	0.9774	0.9856	−-

Note: All items were coded as non-exposed (0) and exposed (1). All associations were significant at p < 0.001 following Bonferroni correction for multiple comparisons.

Table 3 presents the weighted prevalence of ACE exposure and non-response across demographic characteristics. Further analysis suggests that at older age the IRR of ACE exposure is lower (IRR = 0.84, 95 % CI = 0.83–0.85) whereas the opposite pattern was observed with ACE non-response (IRR = 1.04, 95 % CI = 1.00–1.08). Identifying as female was associated with an elevated rate of ACE exposure compared to males (IRR = 1.22, 95 % CI = 1.18–1.25), but was not associated with cumulative missing ACE (IRR = 1.07, 95 % CI = 0.95–1.20). Important trends by race were also observed. Whereas the rate of cumulative ACE was lower among those identifying as non-Hispanic Black (IRR = 0.91, 95 % CI = 0.87–0.96) or Asian (IRR = 0.67, 95 % CI = 0.56–0.77) than among non-Hispanic Whites, the opposite pattern emerged for cumulative missing ACE (non-Hispanic Black: IRR = 1.50, 95 % CI = 1.26–1.77; Asian: IRR = 1.67, 95 % CI = 1.14–2.46). Similarly, there was no association between the rate of ACE exposure and identifying as Hispanic (IRR = 1.00, 95 % CI = 0.94–1.08), but identifying as Hispanic was associated with a higher rate of missing ACE (IRR = 1.80, 95 % CI = 1.42–2.27). Finally, respondents of other/mixed race identities reported a higher rate of ACE exposure (IRR = 1.46, 95 % CI = 1.36–1.56) and missing ACE (IRR = 1.49, 95 % CI = 1.14–1.93) than other groups.

**Table 3:** Prevalence of ACE Exposure and Declined to Respond across Demographic Characteristics in the 2021 Behavioral Risk Factor Surveillance Survey (unweighted n = 58,278).

	Unweighted N	% ACE Exposed			% ACE Declined		
Characteristic		0	1–3	4+	0	1–3	4+
Age (years)							
*18*–*24*	2,880	24.55	44.27	31.19	87.52	10.29	2.2
*25*–*34*	4,946	23.79	44.43	31.79	87.17	10.38	2.45
*35*–*44*	6,674	28.48	45.09	26.43	87.93	9.94	2.13
*45*–*54*	8,182	33.8	44.14	22.06	86.92	10.32	2.77
*55*–*64*	11,088	36.2	44.83	18.97	88.32	9.08	2.6
*65 and older*	24,508	48.66	42.06	9.28	88.84	8.75	2.41
							
Sex at birth							
*Female*	32,089	33.91	41.53	24.56	87.28	10.3	2.43
*Male*	26,189	35.0	46.56	18.44	88.57	8.99	2.44
							
Race							
*NH White*	46,026	35.3	43.51	21.19	89.96	8.06	1.98
*NH Black*	5,905	32.57	47.71	19.72	81.9	15.17	2.93
*NH Asian*	689	44.39	40.59	15.02	85.95	10.98	3.06
*Hispanic*	2,418	29.33	45.24	25.43	85.97	10.18	3.85
*Other/Mixed*	2,067	22.09	41.03	36.88	82.99	14.83	2.17
*Unknown*	1,173	40.25	36.92	22.84	75.02	15.5	9.48
							
Total	58,278	34.43	43.96	21.61	87.9	9.66	2.43

Note: NH = Non-Hispanic. Prevalence estimates calculated using the “svy: tab” command in Stata version 18.

ACE non-response was associated with multiple health indicators after adjusting for demographic covariates (Table 4). Among health behaviors, declining to respond to 1–3 ACE items was associated with an increased odds of smoking (OR = 1.57, 95 % CI = 1.39–1.77) and e-cigarette use (OR = 1.40, 95 % CI = 1.15–1.70) compared to those who answered all items. However, declining to respond to ACE items was not associated with binge drinking or heavy drinking in contrast to the pattern for ACE exposure. Similarly, declining to respond to 1–3 ACE was associated with lower odds of reporting past month exercise (OR = 0.74, 95 % CI = 0.67–0.82) while ACE exposure was not associated with exercising. Declining to respond to 1–3 ACE was associated with higher odds of ever testing for HIV (OR = 1.33, 95 % CI = 1.20–1.47). There was a similar pattern of associations across different health conditions between ACE non-response and ACE exposure. Those declining to respond to 1–3 ACE items were at an increased odds of being underweight (OR = 1.74, 95 % CI = 1.25–2.41), obese (OR = 1.15, 95 % CI = 1.05–1.27), having difficulty concentrating or remembering (OR = 1.96, 95 % CI = 1.74–2.20), or reporting fair or poor self-reported health (OR = 1.56, 95 % CI = 1.39–1.74). Declining to respond to four or more ACE items was associated with difficulty concentrating or remembering (OR = 1.47, 95 % CI = 1.13–1.90), or reporting fair or poor self-reported health (OR = 1.28, 95 % CI = 1.01–1.63). Finally, declining to respond to 1–3 ACE items was associated with being diagnosed with depression (OR = 1.49, 95 % CI = 1.34–1.66), diabetes (OR = 1.21, 95 % CI = 1.06–1.40), heart attack (OR = 1.37, 95 % CI = 1.13–1.67), high blood pressure (OR = 1.16, 95 % CI = 1.05–1.28), and stroke (OR = 1.43, 95 % CI = 1.14–1.79; 4 + declined: OR = 1.94, 95 % CI = 1.16–3.24). Supplemental analyses were completed with the 2020 BRFSS data (Supp Tables 1-4) and results were generally consistent.

**Table 4:** Adjusted associations between ACE exposure (reference = none) and declined ACE (reference = none) with health indicators in the 2021 Behavioral Risk Factor Surveillance Survey (unweighted n = 58,278).

	ACE Exposed				ACE Declined			
Health indicator	1–3		4+		1–3		4+	
	OR	95 % CI	OR	95 % CI	OR	95 % CI	OR	95 % CI
*Health behaviors*								
Smoking	**1.66**	**1.51**–**1.83**	**3.32**	**2.99**–**3.68**	**1.57**	**1.39**–**1.77**	1.11	0.86–1.44
E-cigarette use	**1.79**	**1.51**–**2.21**	**3.31**	**2.79**–**3.94**	**1.40**	**1.15**–**1.70**	0.85	0.51–1.44
Smokeless tobacco	0.97	0.84–1.13	1.00	0.80–1.23	1.09	0.84–1.40	0.69	0.41–1.17
								
Binge drinking	**1.41**	**1.29**–**1.55**	**1.72**	**1.54**–**1.91**	0.96	0.83–1.10	0.70	0.47–1.04
Heavy drinking	**1.50**	**1.32**–**1.71**	**1.94**	**1.67**–**2.24**	0.96	0.79–1.16	0.77	0.47–1.25
								
Exercise	1.01	0.94–1.08	0.94	0.86–1.02	**0.74**	**0.67**–**0.82**	0.91	0.74–1.11
								
Ever tested for HIV	**1.59**	**1.49**–**1.70**	**3.02**	**2.78**–**3.28**	**1.33**	**1.20**–**1.47**	1.11	0.90–1.37
								
*Health conditions*								
Underweight	0.97	0.74–1.26	1.04	0.77–1.41	**1.74**	**1.25**–**2.41**	1.16	0.62–2.17
Obese	**1.19**	**1.12**–**1.27**	**1.54**	**1.42**–**1.67**	**1.15**	**1.05**–**1.27**	0.87	0.70–1.09
								
Difficulty concentrating or remembering	**1.76**	**1.58**–**1.95**	**4.73**	**4.23**–**5.27**	**1.96**	**1.74**–**2.20**	**1.47**	**1.13**–**1.90**
								
Fair/poor self-rated health	**1.29**	**1.19**–**1.39**	**2.53**	**2.30**–**2.79**	**1.56**	**1.39**–**1.74**	**1.28**	**1.01**–**1.63**
								
*Diagnoses*								
Told depression	**2.14**	**1.97**–**2.33**	**5.32**	**4.83**–**5.85**	**1.49**	**1.34**–**1.66**	1.09	0.86–1.39
Told diabetes	**1.16**	**1.06**–**1.26**	**1.50**	**1.34**–**1.67**	**1.21**	**1.06**–**1.40**	1.09	0.83–1.45
Told heart attack	1.11	0.98–1.25	**1.86**	**1.59**–**2.18**	**1.37**	**1.13**–**1.67**	1.23	0.81–1.85
Told high blood pressure	**1.12**	**1.05**–**1.19**	**1.30**	**1.20**–**1.42**	**1.16**	**1.05**–**1.28**	0.85	0.70–1.04
Told high cholesterol	**1.20**	**1.12**–**1.28**	**1.51**	**1.39**–**1.65**	1.07	0.96–1.18	0.84	0.66–1.06
Told stroke	1.10	0.95–1.26	**1.75**	**1.46**–**2.09**	**1.43**	**1.14**–**1.79**	**1.94**	**1.16**–**3.24**

Note: Bold values signify significant associations. Models adjusted for age, sex, and race/ethnicity.

## 6 Discussion

In most cases, the pattern of associations between declining to respond to ACE items and health indicators were similar to the associations between respondents who provide full information on all ACE items. Declining to respond to ACE items on surveys or screening measures can stem from the sensitive nature of the experiences being assessed, concerns about privacy and trust, psychological distress, avoiding reminders of earlier trauma, and various societal and cultural factors. Recognizing and addressing these potential barriers is crucial for ensuring the validity and reliability of ACE data and facilitating and promoting an environment that encourages individuals to disclose their experiences in a safe and supportive manner.

ACE non-response was associated with a decreased odds of past month exercise; however, ACE non-response was associated with an increased odds of lifetime HIV testing. While we cannot make specific conclusions regarding ACE non-response and health promoting behaviors, we did note similarities to ACE exposed respondents. It is possible that trauma-exposed individuals have developed coping strategies or engaged in protective factors that contribute to healthier lifestyles (Rogers et al., 2023). Public health interventions should consider these protective factors and explore how to promote healthy behaviors among individuals who have experienced ACE, but may be less likely to disclose them. On the other hand, the increased odds of cigarette and electronic cigarette use among ACE non-responders suggest the potential that non-responders are as likely to adopt certain maladaptive coping behaviors as individuals who provide full information—though this is not consistent across all adverse health behaviors (e.g., binge drinking). Individuals who withhold information about ACE may be even more likely to engage in specific substance use behavior to alleviate emotional and psychological distress linked to adverse experiences (Rogers et al., 2022), but longitudinal work is needed to confirm this possibility. It is also plausible that the health consequences and health compromising behaviors associated with non-response to specific items are driven by the ACE items that were answered affirmatively in the survey. Our findings underscore the importance of further research on the benefits of targeted interventions to address maladaptive behaviors such as substance use and provide alternative, healthier coping strategies for individuals affected by ACE.

The finding that individuals who decline to respond to ACE items have increased odds of being underweight or obese is particularly concerning. Addressing disordered eating behaviors or unhealthy weight management practices aligns with extant research on ACE exposure and weight status (Davis et al., 2019, Rienecke et al., 2022, Schroeder et al., 2021), and practitioners should consider interventions for those who decline to respond to ACE questionnaires. Public health initiatives should focus on how to incorporate trauma informed practices into programs that promote healthy body image and weight management prevention and intervention programs. The association between ACE non-response and the increased odds of experiencing difficulty concentrating or remembering raises further concern about the potential cognitive impact of ACE (Ward et al., 2022). Individuals who choose not to disclose ACE may be more likely to face challenges related to cognitive function than individuals who do disclose, which have implications for educational attainment, employment opportunities, and overall quality of life. Future work is needed to develop resources to support trauma exposed populations and address cognitive difficulties among individuals affected by ACE, regardless of disclose status. Non-disclosure of ACE is also associated with a subjective perception of poorer health status which is correlated with both ACE exposure and other health problems (Chartier et al., 2010, Felitti et al., 1998).

Declining to respond to ACE items was associated with self-reported diagnoses of depression, diabetes, heart attack, high blood pressure, and stroke. These findings were remarkably similar to the long-term health consequences associated with ACE among those who acknowledge these experiences. Researchers and practitioners should be aware that individuals who do not disclose ACE may be at risk for similar health outcomes as their counterparts with varying levels of ACE exposure. Thus, increased awareness, early detection, and appropriate interventions for individuals affected by ACE, even if they do not disclose them, are a priority. As clinicians consider the role of ACE screening in primary care settings (Campbell, 2020), attention is needed to develop strategies to reduce skipping ACE items on screening forms. In the same vein, the increased odds of individuals who declined to respond to ACE items ever being tested for HIV implies those with unknown histories of family trauma may have different reasons for seeking specific preventive healthcare services (Grigsby et al., 2022). There is a critical need to address barriers and improve healthcare utilization for ACE exposed populations.

It is equally important to recognize individual reasons for not responding to ACE items. It is particularly significant when considering marginalized communities, such as racial or ethnic minorities, who may have historical or systemic mistrust toward medical or research institutions. Mistrust could partially explain the observed racial/ethnic differences in missing ACE responses. Further exploration is warranted to discern whether these associations stem from individual trauma exposures or larger systemic issues inherent to the collection of sensitive data. Additionally, language barriers, limited health literacy, or a constrained understanding of the survey or screening process can also impede accurate and comprehensive responses.

There are several limitations to this study. First, this secondary analysis cannot support causal conclusions or identify reasons/motivations why individuals marked “don’t know” or “refuse” to ACE items and there are several potential explanations. One reason may be that individuals may be unsure what is being asked or the survey item may have been ambiguous in its presentation of ACE. Other demographic variables (such as education level) could partially explain these results and should be assessed in future investigations. Second, questions about this sensitive topic may illicit discomfort, especially if perceived as intrusive and therefore are skipped. Third, participants might avoid disclosing information that could reflect negatively on them (i.e., social desirability bias). Fourth, due to wording of the neglect items in the BRFSS, we did not include these items in our analysis. Given the length of surveillance surveys such as the BRFSS, participants could experience fatigue or lack of motivation to continue or cognitive overload where the participant becomes overwhelmed by the volume/complexity of questions. As a result, this work may not generalize to shorter screening measures such as those used in healthcare settings. Lastly, the sample size of participants with four or more missing ACE responses was small and our analysis was of this subgroup was underpowered. This finding also warrants further exploration as it suggests individuals may only be skipping singular, and personally significant, experiences of childhood trauma which may partially explain our consistent findings between declining to respond to 1–3 ACE items and multiple health indicators in this sample.

These findings have concrete implications for future public health research and practice. Public and clinical health professionals should explore methods to confirm experiences of ACE in screening (Barnett et al., 2021). Exploring how non-response to specific ACE items may vary depending on the mode of administration (e.g., phone interview versus paper questionnaire) and level of anonymity can offer insights into the effectiveness and reliability of different ACE assessment approaches. Further, efforts to integrate the principles of trauma-informed care into early intervention and targeted public health interventions for individuals affected by ACE, irrespective of their disclosure status, is needed. This can involve creating environments that prioritize safety, trust, and empowerment, integrating trauma-informed care principles into program design and implementation, and addressing the broader socio-environmental context in which ACE-associated health disparities arise.

## Funding

The authors received no funding to complete this work.

## CRediT authorship contribution statement

**Timothy J. Grigsby:** Writing – original draft, Project administration, Methodology, Formal analysis, Conceptualization. **Madalyn Larson:** Writing – review & editing, Validation, Software, Formal analysis. **Andrea Lopez:** Writing – review & editing, Writing – original draft. **Sarah Sharmin:** Writing – review & editing. **Ying Guo:** Writing – review & editing. **Myriam Forster:** Writing – review & editing. **Sheniz Moonie:** Writing – review & editing.

## Declaration of competing interest

The authors declare that they have no known competing financial interests or personal relationships that could have appeared to influence the work reported in this paper.

## Data Availability

Data will be made available on request.

## References

[b0005] *ACEs Aware*. (n.d.). ACEs Aware. Retrieved July 22, 2023, from https://www.acesaware.org/.

[b0010] Alcalá H.E., von Ehrenstein O.S., Tomiyama A.J. (2016). Adverse Childhood Experiences and Use of Cigarettes and Smokeless Tobacco Products. J. Community Health.

[b0015] Anda R.F., Felitti V.J., Bremner J.D., Walker J.D., Whitfield Ch., Perry B.D., Dube S.R., Giles W.H. (2006). The enduring effects of abuse and related adverse experiences in childhood. Eur. Arch. Psychiatry Clin. Neurosci..

[b0020] Barnett M.L., Sheldrick R.C., Liu S.R., Kia-Keating M., Negriff S. (2021). Implications of adverse childhood experiences screening on behavioral health services: A scoping review and systems modeling analysis. Am. Psychol..

[b0025] California Department of Health Care Services. (n.d.). *ACEs Aware Master FAQ*. Retrieved July 20, 2023, from https://www.acesaware.org/wp-content/uploads/2019/11/ACEs-Aware-Master-FAQ.pdf.

[b0030] Campbell T.L. (2020). Screening for Adverse Childhood Experiences (ACEs) in Primary Care: A Cautionary Note. J. Am. Med. Assoc..

[b0035] *CDC - BRFSS*. (2021, October 8). https://www.cdc.gov/brfss/index.html.

[b0040] CDC. (2023, June 29). *Fast Facts: Preventing Adverse Childhood Experiences |Violence Prevention|Injury Center|CDC*. https://www.cdc.gov/violenceprevention/aces/fastfact.html.

[b0045] Chartier M.J., Walker J.R., Naimark B. (2010). Separate and cumulative effects of adverse childhood experiences in predicting adult health and health care utilization. Child Abuse Negl..

[b0050] Crouch E., Strompolis M., Bennett K.J., Morse M., Radcliff E. (2017). Assessing the interrelatedness of multiple types of adverse childhood experiences and odds for poor health in South Carolina adults. Child Abuse Negl..

[b0055] Danese, A., & J Lewis, S. (2017). Psychoneuroimmunology of Early-Life Stress: The Hidden Wounds of Childhood Trauma? *Neuropsychopharmacology*, *42*(1), Article 1. Doi: 10.1038/npp.2016.198. 10.1038/npp.2016.198 PMC5143500 27629365

[b0060] Danese A., McEwen B.S. (2012). Adverse childhood experiences, allostasis, allostatic load, and age-related disease. Physiol. Behav..

[b0065] Davis L., Barnes A.J., Gross A.C., Ryder J.R., Shlafer R.J. (2019). Adverse Childhood Experiences and Weight Status among Adolescents. J. Pediatr..

[b0070] Downing N.R., Akinlotan M., Thornhill C.W. (2021). The impact of childhood sexual abuse and adverse childhood experiences on adult health related quality of life. Child Abuse Negl..

[b0075] Fang L., Chuang D.-M., Lee Y. (2016). Adverse childhood experiences, gender, and HIV risk behaviors: Results from a population-based sample. Prev. Med. Rep..

[b0080] Felitti V.J., Anda R.F., Nordenberg D., Williamson D.F., Spitz A.M., Edwards V., Koss M.P., Marks J.S. (1998). Relationship of Childhood Abuse and Household Dysfunction to Many of the Leading Causes of Death in Adults: The Adverse Childhood Experiences (ACE) Study. Am. J. Prev. Med..

[b0085] Finkelhor D., Shattuck A., Turner H., Hamby S. (2013). Improving the Adverse Childhood Experiences Study Scale. JAMA Pediatr..

[b0090] Grigsby T.J., Rogers C.J., Albers L.D., Benjamin S.M., Forster M. (2022). Adverse childhood experiences and college student health promotion outcomes: Another perspective on the trauma-health relationship. Child Abuse Rev..

[b0095] Hajat A., Nurius P., Song C. (2020). Differing trajectories of adversity over the life course: Implications for adult health and well-being. Child Abuse Negl..

[b0100] Hall T., Rooks R., Kaufman C. (2020). Intersections of Adverse Childhood Experiences, Race and Ethnicity and Asthma Outcomes: Findings from the Behavioral Risk Factor Surveillance System. Int. J. Environ. Res. Public Health.

[b0105] Hardt J., Rutter M. (2004). Validity of adult retrospective reports of adverse childhood experiences: Review of the evidence. J. Child Psychol. Psychiat..

[b0110] Lee R.D., Chen J. (2017). Adverse childhood experiences, mental health, and excessive alcohol use: Examination of race/ethnicity and sex differences. Child Abuse Negl..

[b0115] Loudermilk E., Loudermilk K., Obenauer J., Quinn M.A. (2018). Impact of adverse childhood experiences (ACEs) on adult alcohol consumption behaviors. Child Abuse Negl..

[b0120] Mendoza I.D., Banda J.A., Giano Z., Hubach R.D. (2023). Association between adverse childhood experiences and fruit and vegetable intake among a national sample of U.S. adults. Prev. Med. Rep..

[b0125] Merrick M.T., Ford D.C., Ports K.A., Guinn A.S., Chen J., Klevens J., Metzler M., Jones C.M., Simon T.R., Daniel V.M., Ottley P., Mercy J.A. (2019). Vital Signs: Estimated Proportion of Adult Health Problems Attributable to Adverse Childhood Experiences and Implications for Prevention — 25 States, 2015–2017. MMWR Morb. Mortal. Wkly Rep..

[b0130] Mwachofi A., Imai S., Bell R.A. (2020). Adverse childhood experiences and mental health in adulthood: Evidence from North Carolina. J. Affect. Disord..

[b0135] Rienecke R.D., Johnson C., Le Grange D., Manwaring J., Mehler P.S., Duffy A., McClanahan S., Blalock D.V. (2022). Adverse childhood experiences among adults with eating disorders: Comparison to a nationally representative sample and identification of trauma profiles. J. Eat. Disord..

[b0140] Rogers E.M., Banks N.F., Tomko P.M., Sciarrillo C.M., Emerson S.R., Thomas E.B.K., Taylor A., Teague T.K., Jenkins N.D.M. (2023). Progressive exercise training improves cardiovascular psychophysiological outcomes in young adult women with a history of adverse childhood experiences. J. Appl. Physiol..

[b0145] Rogers C.J., Pakdaman S., Forster M., Sussman S., Grigsby T.J., Victoria J., Unger J.B. (2022). Effects of multiple adverse childhood experiences on substance use in young adults: A review of the literature. Drug Alcohol Depend..

[b0150] Rudenstine S., Espinosa A., McGee A.B., Routhier E. (2019). Adverse childhood events, adult distress, and the role of emotion regulation. Traumatology.

[b0155] Schroeder K., Schuler B.R., Kobulsky J.M., Sarwer D.B. (2021). The association between adverse childhood experiences and childhood obesity: A systematic review. Obes. Rev..

[b0160] Sonu S., Post S., Feinglass J. (2019). Adverse childhood experiences and the onset of chronic disease in young adulthood. Prev. Med..

[b0165] Ward K., Ryan-Ibarra S., Smith M., Sanchez-Vaznaugh E.V. (2022). Adverse childhood experiences and cognitive disability in the 2019 United States behavioral risk factor surveillance system. Prev. Med. Rep..

[b0170] Wiest-Stevenson C., Lee C. (2016). Trauma-Informed Schools. J. Eviden.-Informed Soc. Work.

