# Supplementary material for: Non-response to ACE items is associated with demographic variables and health indicators in the 2021 Behavioral Risk Factor Surveillance System
Source: Prev Med Rep. 2024 May 3;42:102749. doi: 10.1016/j.pmedr.2024.102749 (PMC11089347; doi:10.1016/j.pmedr.2024.102749)
Supplement: Supplementary Tables [file mmc1.docx]

Supp Table 1. Prevalence of ACE Exposure and Declined to Respond by item in the 2020 Behavioral Risk Factor Surveillance Survey* (unweighted n = 128,496)

| Item | % Exposed | % Declined |
| --- | --- | --- |
| Did you live with anyone who was depressed, mentally ill, or suicidal? | 16.82 | 1.74 |
| Did you live with anyone who was a problem drinker or alcoholic? | 22.73 | 1.32 |
| Did you live with anyone who used illegal street drugs or who abused prescription medications? | 11.52 | 1.4 |
| Did you live with anyone who served time or was sentenced to serve time in a prison, jail, or other correctional facility? | 9.57 | 1.21 |
| Were your parents separated or divorced? | 30.17 | 3.84 |
| How often did your parents or adults in your home ever slap, hit, kick, punch or beat each other up? | 16.62 | 2.89 |
| Not including spanking, (before age 18), how often did a parent or adult in your home ever hit, beat, kick, or physically hurt you in any way? | 23.40 | 2.14 |
| How often did a parent or adult in your home ever swear at you, insult you, or put you down? | 32.57 | 2.56 |
| How often did anyone at least 5 years older than you or an adult, ever touch you sexually? | 11.16 | 2.33 |
| How often did anyone at least 5 years older than you or an adult, try to make you touch them sexually? | 8.23 | 2.3 |
| How often did anyone at least 5 years older than you or an adult, force you to have sex? | 5.20 | 2.12 |

Note: Prevalence estimates were calculated using the “svy: tab” command in Stata version 17.

States

*States included in the 2020 ACE Module: Alabama, Arizona, District of Columbia, Florida, Georgia, Hawaii, Idaho, Iowa, Kentucky, Mississippi, Missouri, Montana, Nevada, North Dakota, Rhode Island, South Carolina, South Dakota, Texas, Utah, Virginia, Wisconsin, and Wyoming

Table 2. Prevalence of ACE Exposure and Declined to Respond across Demographic Characteristics in the 2020 Behavioral Risk Factor Surveillance Survey* (unweighted n = 128,496)

|  | Unweighted N | % ACE Exposed | | | % ACE Declined | | |
| --- | --- | --- | --- | --- | --- | --- | --- |
| Characteristic |  | 0 | 1-3 | 4+ | 0 | 1-3 | 4+ |
| Age (years) |  |  |  |  |  |  |  |
| *18-24* | 7,788 | 28.73 | 45.02 | 26.24 | 89.79 | 9.03 | 1.18 |
| *25-34* | 13,042 | 27.85 | 45.46 | 26.69 | 88.41 | 9.89 | 1.7 |
| *35-44* | 16,383 | 31.84 | 43.19 | 24.98 | 88.96 | 9.38 | 1.66 |
| *45-54* | 18,763 | 34.71 | 43.37 | 21.92 | 89.31 | 9.16 | 1.53 |
| *55-64* | 24,456 | 37.34 | 44.81 | 17.85 | 90.47 | 8.1 | 1.42 |
| *65 and older* | 48,064 | 49.27 | 41.91 | 8.82 | 90.05 | 8.74 | 1.21 |
|  |  |  |  |  |  |  |  |
| Sex at birth |  |  |  |  |  |  |  |
| *Female* | 70,488 | 36.55 | 41.16 | 22.29 | 89.34 | 9.28 | 1.38 |
| *Male* | 58,008 | 36.17 | 46.63 | 17.2 | 89.78 | 8.71 | 1.51 |
|  |  |  |  |  |  |  |  |
| Race |  |  |  |  |  |  |  |
| *NH White* | 94,086 | 36.47 | 43.52 | 20.01 | 91.41 | 7.36 | 1.23 |
| *NH Black* | 11,301 | 32.42 | 48.14 | 19.44 | 81.88 | 16.53 | 1.59 |
| *NH Asian* | 3,414 | 54.15 | 38.99 | 6.86 | 90.94 | 7.69 | 1.37 |
| *Hispanic* | 9,909 | 37.09 | 42.73 | 20.17 | 90.51 | 8 | 1.49 |
| *Other/Mixed* | 7,449 | 26.94 | 42.08 | 30.98 | 86.28 | 11.15 | 2.57 |
| *Unknown* | 2,337 | 41.33 | 40.04 | 18.63 | 78.42 | 16.19 | 5.39 |
|  |  |  |  |  |  |  |  |
| Total | 128,476 | 36.49 | 43.36 | 20.15 | 88.25 | 9.94 | 1.8 |

Note: NH = Non-Hispanic. Prevalence estimates calculated using the “svy: tab” command in Stata version 17.

*States included in the 2020 ACE Module: Alabama, Arizona, District of Columbia, Florida, Georgia, Hawaii, Idaho, Iowa, Kentucky, Mississippi, Missouri, Montana, Nevada, North Dakota, Rhode Island, South Carolina, South Dakota, Texas, Utah, Virginia, Wisconsin, and Wyoming

Table 3. Tetrachoric associations between exposure to individual ACE (above dashed line) and declined to answer individual ACE* (below dashed line). (unweighted n = 128,496)

|  | 1 | 2 | 3 | 4 | 5 | 6 | 7 | 8 | 9 | 10 | 11 |
| --- | --- | --- | --- | --- | --- | --- | --- | --- | --- | --- | --- |
| 1. Did you live with anyone who was depressed, mentally ill, or suicidal? | -- | 0.5089 | 0.6232 | 0.4884 | 0.3678 | 0.4759 | 0.4418 | 0.5546 | 0.4644 | 0.4543 | 0.4465 |
| 1. Did you live with anyone who was a problem drinker or alcoholic? | 0.9333 | -- | 0.6251 | 0.5563 | 0.4279 | 0.6004 | 0.4242 | 0.4932 | 0.3953 | 0.3898 | 0.4031 |
| 1. Did you live with anyone who used illegal street drugs or who abused prescription medications? | 0.9185 | 0.9576 | -- | 0.7414 | 0.4546 | 0.5064 | 0.4207 | 0.5037 | 0.4216 | 0.4344 | 0.4339 |
| 1. Did you live with anyone who served time or was sentenced to serve time in a prison, jail, or other correctional facility? | 0.9367 | 0.9676 | 0.9714 | -- | 0.4690 | 0.4845 | 0.3722 | 0.4267 | 0.3664 | 0.3781 | 0.3930 |
| 1. Were your parents separated or divorced? | 0.7883 | 0.8544 | 0.8504 | 0.8924 | -- | 0.4719 | 0.3098 | 0.3575 | 0.3049 | 0.3187 | 0.3170 |
| 1. How often did your parents or adults in your home ever slap, hit, kick, punch or beat each other up? | 0.8170 | 0.8763 | 0.8708 | 0.9021 | 0.7394 | -- | 0.6381 | 0.6101 | 0.4462 | 0.4354 | 0.4542 |
| 1. Not including spanking, (before age 18), how often did a parent or adult in your home ever hit, beat, kick, or physically hurt you in any way? | 0.8482 | 0.8992 | 0.8941 | 0.9240 | 0.7801 | 0.8774 | -- | 0.6481 | 0.4582 | 0.4505 | 0.4797 |
| 1. How often did a parent or adult in your home ever swear at you, insult you, or put you down? | 0.8217 | 0.8778 | 0.8684 | 0.9048 | 0.7299 | 0.8323 | 0.9014 | -- | 0.4675 | 0.4646 | 0.4748 |
| 1. How often did anyone at least 5 years older than you or an adult, ever touch you sexually? | 0.8410 | 0.8921 | 0.8892 | 0.9202 | 0.7656 | 0.8266 | 0.8817 | 0.8552 | -- | 0.9300 | 0.8803 |
| 1. How often did anyone at least 5 years older than you or an adult, try to make you touch them sexually? | 0.8424 | 0.8965 | 0.8930 | 0.9215 | 0.7714 | 0.8278 | 0.8821 | 0.8550 | 0.985 | -- | 0.8711 |
| 1. How often did anyone at least 5 years older than you or an adult, force you to have sex? | 0.8419 | 0.8970 | 0.8954 | 0.9238 | 0.7763 | 0.8256 | 0.8803 | 0.8566 | 0.972 | 0.9804 | -- |

Note: All associations were significant at p<0.001 following Bonferroni correction for multiple comparisons.

*States included in the 2020 ACE Module: Alabama, Arizona, District of Columbia, Florida, Georgia, Hawaii, Idaho, Iowa, Kentucky, Mississippi, Missouri, Montana, Nevada, North Dakota, Rhode Island, South Carolina, South Dakota, Texas, Utah, Virginia, Wisconsin, and Wyoming

Table 4. Adjusted associations between ACE exposure and declined ACE with health indicators in the 2020 Behavioral Risk Factor Surveillance Survey* (unweighted n = 128,496).

|  | ACE Exposed | | | | ACE Declined | | | |
| --- | --- | --- | --- | --- | --- | --- | --- | --- |
| Health indicator | 1-3 | | 4+ | | 1-3 | | 4+ | |
|  | OR | 95% CI | OR | 95% CI | OR | 95% CI | OR | 95% CI |
| *Health behaviors* |  |  |  |  |  |  |  |  |
| Binge drinking | 1.40 | 1.27-1.54 | 1.70 | 1.52-1.90 | 0.93 | 0.80-1.09 | 0.63 | 0.44-0.90 |
| Heavy drinking | 1.62 | 1.42-1.86 | 2.02 | 1.75-2.34 | 1.09 | 0.87-1.36 | 0.95 | 0.61-1.49 |
|  |  |  |  |  |  |  |  |  |
| Smoking | 1.71 | 1.55-1.88 | 3.17 | 2.86-3.51 | 1.40 | 1.23-1.58 | 1.29 | 0.93-1.77 |
| E-cigarette use | 1.34 | 1.07-1.67 | 1.52 | 1.22-1.90 | 0.89 | 0.69-1.15 | 1.78 | 0.88-3.59 |
| Smokeless tobacco | 1.13 | 0.99-1.30 | 1.23 | 1.05-1.45 | 1.10 | 0.88-1.37 | 0.89 | 0.58-1.40 |
|  |  |  |  |  |  |  |  |  |
| Exercise | 0.92 | 0.85-0.99 | 0.83 | 0.75-0.90 | 0.74 | 0.66-0.83 | 0.85 | 0.64-1.12 |
|  |  |  |  |  |  |  |  |  |
| Ever tested for HIV | 1.39 | 1.30-1.49 | 2.67 | 2.45-2.90 | 1.29 | 1.16-1.43 | 1.22 | 0.93-1.59 |
|  |  |  |  |  |  |  |  |  |
| *Health conditions* |  |  |  |  |  |  |  |  |
| Underweight | 0.94 | 0.76-1.16 | 0.86 | 0.65-1.15 | 1.17 | 0.88-1.57 | 1.27 | 0.74-2.17 |
| Obese | 1.21 | 1.13-1.30 | 1.43 | 1.32-1.56 | 1.22 | 1.09-1.35 | 1.29 | 0.96-1.74 |
|  |  |  |  |  |  |  |  |  |
| Difficulty concentrating or remembering | 1.69 | 1.52-1.89 | 4.80 | 4.29-5.37 | 2.00 | 1.76-2.28 | 1.22 | 0.91-1.62 |
|  |  |  |  |  |  |  |  |  |
| Fair/poor self-rated health | 1.37 | 1.26-1.49 | 2.55 | 2.32-2.81 | 1.41 | 1.27-1.58 | 1.20 | 0.92-1.56 |
|  |  |  |  |  |  |  |  |  |
| Told depression | 2.10 | 1.93-2.28 | 5.63 | 5.13-6.18 | 1.59 | 1.42-1.77 | 1.25 | 0.98-1.60 |
| Told diabetes | 1.13 | 1.04-1.24 | 1.39 | 1.24-1.55 | 0.99 | 0.86-1.14 | 0.94 | 0.71-1.25 |
| Told heart attack | 1.16 | 1.03-1.31 | 1.85 | 1.59-2.16 | 1.16 | 0.98-1.36 | 1.09 | 0.76-1.56 |
| Told stroke | 1.18 | 1.02-1.35 | 1.94 | 1.66-2.28 | 1.37 | 1.15-1.64 | 0.96 | 0.64-1.42 |

*States included in the 2020 ACE Module: Alabama, Arizona, District of Columbia, Florida, Georgia, Hawaii, Idaho, Iowa, Kentucky, Mississippi, Missouri, Montana, Nevada, North Dakota, Rhode Island, South Carolina, South Dakota, Texas, Utah, Virginia, Wisconsin, and Wyoming. Items regarding high blood pressure and high cholesterol were not included in the 2020 BRFSS data.
